# Surgical castration in large dogs: is closed castration safer than open?

**DOI:** 10.18849/ve.v11i3.739

**Published:** 2026-08-10

**Authors:** Florine Morrison

**Keywords:** CANINE, CASTRATION, COMPLICATIONS, ORCHIDECTOMY, NEUTERING, STERILISATION

## Abstract

**PICO question:**

In large dogs weighing more than 20 kg undergoing elective orchidectomy, does a closed castration technique result in fewer postoperative complications than an open castration technique?

**Clinical bottom line:**

**Category of research:**

Treatment.

**Number and type of study designs reviewed:**

One randomised, controlled trial was critically appraised.

**Strength of evidence:**

Weak.

**Outcomes reported:**

The study found that dogs weighing between 3 kg and 53 kg having closed castrations suffered fewer complications up to 10 days postoperatively compared to those having open castrations. Weight of the dog was not a significant factor in complication rate following closed versus open castration. The study reported a greater number of large dogs weighing over 20 kg suffering major complications that needed a medical or surgical intervention. However, the population size of these large dogs was too small to evaluate, with any significance, which technique was safer in reducing the risk of major complications.

**Conclusion:**

Regardless of the size of the dog, there is limited, weak evidence that closed castrations lead to fewer postoperative complications compared to open castrations. However, there is insufficient evidence as to whether this applies to large dogs weighing over 20 kg. Furthermore, there is insufficient evidence on whether closed castrations lead to fewer major postoperative complications, defined as those requiring medical or surgical intervention. There is therefore insufficient evidence to justify a change in clinical practice regarding elective castration technique in large dogs. It can be argued that the best available evidence suggests that closed castrations may be safer overall, but further research is needed to evidence this more strongly in large dogs.

How to apply this evidence in practice

The application of evidence into practice should take into account multiple factors, not limited to: individual clinical expertise, patient’s circumstances and owners’ values, country, location or clinic where you work, the individual case in front of you, the availability of therapies and resources.

Knowledge Summaries are a resource to help reinforce or inform decision making. They do not override the responsibility or judgement of the practitioner to do what is best for the animal in their care.

## Clinical scenario

You work in a practice where most vets tend to perform closed castrations on most dogs, benefiting from less tissue trauma and handling, and a slightly reduced surgery time when compared to open castrations. Following the respective bans of XL Bully breed types in England, Wales, Scotland and Northern Ireland, issued across 2023 to 2024, and the introduction of exemption certification requirements for these dogs, there was a rise in the number of large dogs presenting for neutering. You question whether it would be safer to do open castrations to allow for more secure ligature placement due to the large size of their spermatic cords and greater risk for intra- and postoperative haemorrhage.

## The evidence

One randomised, controlled, single-blinded trial was identified as relevant to the PICO question (Hamilton et al., 2014). The study presented weak evidence that dogs of any size undergoing closed castrations had fewer postoperative complications than open castrations. However, the study presented insufficient evidence of whether this held true in large dogs weighing over 20 kg, and whether the same applied to major complications that required medical or surgical intervention in large dogs.

## Summary of the evidence

**Hamilton et al. (2014):** Comparison of postoperative complications in healthy dogs undergoing open and closed orchidectomy **Aim: **To compare the rate and severity of complications between closed and open orchidectomy in dogs.

Population:	Shelter- and privately owned, sexually intact, healthy, male dogs with a mean age of 2 years and a mean (± SD) weight of 20.7 ± 11.93 kg.
Sample size:	73 dogs.
Intervention details:	Dogs were randomly assigned into two groups by tossing a coin: 34 dogs underwent open castration and 39 underwent closed castration. Standardised protocols were used for anaesthesia, asepsis, pre-scrotal open castration or closed castration technique, and recovery. Dogs from both groups were premedicated (acepromazine 0.02 mg/kg; buprenorphine 0.02 mg/kg; meloxicam 0.2 mg/kg) intravenously and anaesthesia was induced using propofol (1–3 mg/kg) and maintained on isoflurane in oxygen. Surgical sites were prepared aseptically using 4% chlorhexidine followed by alcohol. Surgical drapes, surgeon gowns and sterile gloves were used. 61 castrations were performed by experienced veterinarians and 12 by final-year veterinary students assisted by veterinarians. Dogs in the open castration group had the parietal vaginal tunic incised in order to exteriorise each testicle. The vascular cord and ductus deferens were individually ligated and an additional encircling ligature was applied around both structures using polyglactin 910 prior to transection. The tunic, fascia, and skin were sutured. Dogs in the closed castration group had the spermatic cord exteriorised by tearing fat, fascia, and fibrous attachments away from the cord but leaving the parietal vaginal tunic intact. An encircling and a transfixing ligature were applied to the cord prior to transection. The fascia and skin were sutured. Dogs from both groups were discharged the following day with an Elizabethan collar and instructions to rest for 10 days. Dogs were examined for complications daily by their owners up to 10 days postoperatively and at 5 hours, 24 hours, and 10 days by a veterinary nurse blinded to the treatment group. Pain was measured using the Glasgow composite measure pain scale.
Study design:	Randomised, controlled, single-blinded, multi-site clinical trial.
Outcome Studied:	Age, breed, weight, duration of surgery were recorded. Complications were recorded: incisional haemorrhage, incision discharge, incision swelling, incision bruising, incision erythema, incision dehiscence, incision pain, scrotal bruising, scrotal swelling, scrotal pain, pyoderma, diarrhoea, vomiting. Minor complications were defined as self-limiting within 24 hours and not requiring treatment. Major complications were defined by the requirement for medical or surgical intervention.
Main Findings (relevant to PICO question):	Complications (minor and major) were common (42/73 dogs (58%)). Significantly more complications were seen following open castration (24/34 dogs (71%)) compared to closed castration (18/39 dogs (46%)) (P = 0.04; OR: 2.8; 95% CI: 1.06–7.3). Major complications were not common (8/73 dogs (11%)), with 6/34 dogs (18%) following open castration and 2/39 dogs (5%) following closed castration. Of the 8 dogs suffering major complications, 5 were dogs weighing > 20 kg, with 3 having had open castrations, and 2 had closed castrations. Weight was reported not to be a significant factor in the rate of complications observed. No data was presented to support this statement. 1/39 (2.56%) of the dogs in the closed castration group required surgical intervention as a result of the complication. None of the dogs in the open castration group required surgical intervention.
Limitations:	Insufficient power: insufficient large dogs recruited to demonstrate with confidence any difference in rate of complications by weight or conclude on safety of closed technique in large dogs. Insufficient power: difference in complication rate between open and closed castration groups has very wide confidence interval, with the lower range starting at 1.06, which is very close to the null value (indicating no difference between groups). Despite being considered a significant difference (P = 0.04), there is uncertainty in the measured effect. Confounders not addressed: temperament of the dog, undiagnosed health conditions, breed variation.

### Appraisal, application and reflection

Complications following elective castration in dogs are common in small animal practice. A national audit in the UK found 378/1,464 dogs (25.82%) showing a complication as a result of the surgery (RCVS Knowledge, 2025) of which almost half needed a treatment intervention. There are many risk factors, including surgical technique and asepsis, postoperative care and analgesia, as well as breed, health, age, temperament and size of the dog (Pollari & Bonnett, 1996; Pollari et al., 1996; Miller et al., 2018; Watts, 2018; Yaman & Kanca, 2023; Senocak, 2023; Leal et al., 2024). Castration surgery in dogs is well-described using an open or closed technique (Hedlund, 2002; Boothe, 2003; White, 2016) and both techniques are widely used in the UK (Tivers et al., 2005). Some recommend that an open technique is used in large dogs to allow for more secure ligation of the vascular cord (Adin, 2011; Romagnoli et al., 2024), whereas some sources suggest that a closed technique can reduce the incidence of complications (Shivley et al., 2022). Romagnoli et al. (2024) argue that a closed technique is acceptable if secure ligatures are achieved and advise two-pass friction knots. These recommendations are expert opinion, rather than based on evidence. The aim of this Knowledge Summary is to appraise the available evidence in whether a closed castration results in fewer complications than an open castration in large dogs weighing more than 20 kg.

As part of the literature search strategy, studies were excluded if the study population or intervention was not relevant to the PICO question, or if the intervention or outcome was not reported. For the purpose of this Knowledge Summary, a large dog was defined as one weighing more than 20 kg, which aligns to the threshold used by Hamilton et al. (2014). Studies evaluating either open or closed techniques only were also excluded. Although such studies contribute to the evidence around the safety of either technique, they do not allow for direct comparison and can therefore not support conclusions regarding a causal link between technique and outcome.

Only a single study was found to be relevant to the PICO question on a literature search (Hamilton et al., 2014). The study randomly allocated dogs into either the open or closed castration group thereby controlling selection bias, and the investigators measuring the complications were blinded to which technique the dog had undergone thereby controlling measurement bias. As the treatment involved a surgical procedure, it was not possible to blind the surgeons to which technique was performed, which might have allowed treatment bias to occur, but this was otherwise a well-designed study using standardised protocols for all elements of the interventions and measurements. While the authors reported the mean and standard deviation of the weights of the study dogs, the weight range was only published within a conference abstract as ranging from 3.0 to 52.7 kg (Hamilton et al., 2013).

The study demonstrated significantly more complications in dogs undergoing open compared to closed castrations in 73 dogs, reporting an odds ratio of 2.8. However, the 95% confidence interval reported was very wide, with the lower limit at 1.06 being close to an interpretation of no difference between the open and closed techniques. A greater population size would be needed to strengthen the confidence in these findings.

A power calculation, performed at ~80% through the study, had identified the need for an additional 29 dogs. By narrowing their aim, the authors were able to use their smaller population size of 73, but this remained the main limitation to the evidence presented. This meant that the study was unable to compare the severity of complications between closed and open techniques as set out in their aim. It also meant they were unable to demonstrate, with confidence, whether large dogs had lower complication rates following a closed castration technique. The authors state that the weight of the dog did not appear to be a significant factor in the rate of complications observed between open and closed castrations. However, the results to support this statement were not included in the paper. Therefore, there is currently insufficient evidence of a lower complication rate for large dogs undergoing closed versus open castrations.

The authors did find a greater number of large dogs suffering major complications (5/8 of the major complications were dogs weighing more than 20 kg) but the sample size was too small and the difference was not significant. In this study, 2/2 dogs that had a major complication following closed castration weighed more than 20 kg; and 3/6 dogs with major complications following open castration weighed more than 20 kg. It remains possible that either or neither castration technique increases the risk of major complications in large dogs, but the study would need a greater population size to investigate this further.

The severity of postoperative complications is an important factor when applying the evidence for or against a surgical technique in practice. Where the evidence is weak, the reporting of minor complications is less likely to justify a change to clinical practice, whereas the reporting of major complications might justify change because of the potential for harm (Petitti et al., 2009). Some complications may not result from the surgical techniques being compared. For example, vomiting and diarrhea are unlikely related to whether an open or closed technique was used, whereas swelling and haemorrhage are more likely related (Adin, 2011). Improved classifications of surgical complications have been developed in the medical field to support quality improvement (Dindo et al., 2004) to help bridge this gap between evidence and application. A consensus on how to grade postoperative complications to maximize applicability of evidence would be helpful in veterinary literature. Considering that dog castrations are one of the most common surgeries performed in small animal practice (Adin, 2011), even a small reduction in complication rate would have a great welfare impact. The aforementioned national audit on neutering in the UK for 2024 reported a higher complication rate (378/1,464 dogs (25.82%)) following castration (RCVS Knowledge, 2025) compared to that reported over the cumulative period 2005–2023 (3,720/15,958 dogs (23.3%)) (RCVS Knowledge, 2024). This is an important area for quality improvement but progress through evidence-based practice will need the reporting of surgical complications to be more applicable to support clinical practice.

The population used in the study is very comparable to that seen in first opinion practice in the UK. A common confounder in surgical studies on privately-owned dogs is postoperative management in the home environment and temperament of the dog. This study found that all complications were identified up to 24 hours postoperatively, whilst the dogs were still held in a controlled clinic environment. Although hospitalising dogs for up to 24 hours following elective castration is not common practice, it did address the home environment as a potential confounder in this study. The dogs recruited were considered healthy based only on their medical history and physical examination on the day of the castration. The population could therefore have included undiagnosed conditions such as lungworm or bleeding disorders. Temperament, age, and breed remain confounding factors, though the latter two were evenly distributed in the open and closed castration groups.

This Knowledge Summary has found limited, weak evidence to support that closed castrations give rise to fewer postoperative complications in dogs weighing between 3.0–52.7 kg, with a reported odds ratio of 2.8 and a 95% confidence interval of 1.06-7.3. However, it has found insufficient evidence to ascertain, with confidence, that the conclusion still applies to large dogs weighing over 20 kg due to the small population size of the trial. Furthermore, the small population size meant that there was also insufficient evidence as to whether closed castrations lead to fewer major complications, defined as complications needing medical or surgical intervention. There is therefore insufficient evidence to justify a change in clinical practice regarding elective castration technique in large dogs weighing more than 20 kg. However, it can be argued that the current, best available evidence suggests that closed castrations may be safer overall, but further research is needed to evidence this more strongly. As well as the evidence base, the clinical decision on whether to perform an open or closed castration in a large dog must include the veterinary surgeon’s professional skill and experience as well as what is judged to be right for the individual dog.

## Methodology

**Search Strategy:** 

Databases searched and dates covered:	CAB Abstracts accessed via CABI digital library 1973–2025 Week 32PubMed accessed via NCBI website 1946–August 2025
Search strategy:	CAB Abstracts: Dog* OR canine OR canis Castrat* OR neuter OR orchidectomy OR orchiectomy OR gonadectom* OR steriliz* OR sterilis* (open OR closed) ) 1 and 2 and 3 PubMed:(dog OR dogs or canine OR canis) AND (castrat* OR neuter OR orchidectomy OR orchiectomy OR gonadectomy* OR steriliz* OR sterilis*) AND ((open OR closed)
Dates searches performed:	07 August 2025

**Exclusion / Inclusion Criteria:** 

Exclusion:	Not relevant to PICO question. Not primary research article. Not in English language.
Inclusion:	Experimental studies. Observational studies.

**Search Outcome:** 

Database	Number of results	Excluded – not relevant to PICO	Excluded – not primary research	Excluded – surgical technique not reported	Excluded – not in English	Total relevant papers
CAB Abstracts	467	462	1	2	1	1
PubMed	158	156	0	1	0	1
Total relevant papers when duplicates removed						1

## Acknowledgements

The author would like to thank the academic support at the University of Liverpool Veterinary Postgraduate Unit for helpful comments in the preparation of this manuscript.

## ORCiD

Florine Morrison: 0009 0008 5810 0027

## Disclaimer

Knowledge Summaries are a peer-reviewed article type which aims to answer a clinical question based on the best available current evidence. It does not override the responsibility of the practitioner. Informed decisions should be made by considering such factors as individual clinical expertise and judgement along with patient’s circumstances and owners’ values. Knowledge Summaries are a resource to help inform and any opinions expressed within the Knowledge Summaries are the author's own and do not necessarily reflect the view of the RCVS Knowledge. Authors are responsible for the accuracy of the content. While the Editor and Publisher believe that all content herein are in accord with current recommendations and practice at the time of publication, they accept no legal responsibility for any errors or omissions, and make no warranty, express or implied, with respect to material contained within. For further information please refer to our Terms of Use.
